# The Influence of Acute Diseases upon Insanity

**Published:** 1902-04-19

**Authors:** 


### THE INFLUENCE OF ACUTE DISEASES UPON INSANITY.

In the course of a clinical lecture delivered a week or two ago at the Polyclinic by Mr. Jonathan Hutchinson we saw a case of leprosy in which great improvement had taken place during the progress of an attack of measles, and Mr. Hutchinson incidentally remarked upon the interesting fact that the occurrence of any of the exanthemata seemed for a time to modify the course of this strange disease. The same thing has been often noted in regard to other maladies, and just now a paper appears from the pen of Dr. Warren,[1] of the Long Island State Hospital, in which he deals with the influence of acute disease upon insanity. He says it is a well-known fact that any acute inflammatory condition arising during the course of an acute mental disease often alleviates the condition, and in many cases seems to have been instrumental in bringing about a complete recovery from insanity. Of the infectious diseases which seem to exercise the greatest influence for good in this respect he particularly notices typhoid fever and erysipelas, but suppurations, gangrenous conditions, fractures, dislocations, etc., have also had in many cases a beneficial effect. Dr. Warren says that patients suffering from mania appear to improve more frequently during and after an attack of infectious disease than those suffering from other forms of insanity. Cases of paresis and other instances of degeneration of nerve cells and fibres never show any improvement. As to the cause of the improvement it seems fair to believe that the toxins generated during the course of an infectious disease counteract in some degree those to which the existing maniacal condition is due, and thus the irritation of the nerve cells is lessened. Some cases, however, do not improve, and this may be due to the nerve cells having already suffered so much from the effects of the poison that they do not recover on its neutralisation. Some striking cases are related.

[1] Brooklyn Med. Jour., April.

